# Dopamine facilitates the translation of physical exertion into assessments of effort

**DOI:** 10.1038/s41531-023-00490-4

**Published:** 2023-04-01

**Authors:** Purnima Padmanabhan, Agostina Casamento-Moran, Aram Kim, Anthony J. Gonzalez, Alexander Pantelyat, Ryan T. Roemmich, Vikram S. Chib

**Affiliations:** 1 Department of Neuroscience, Johns Hopkins School of Medicine, Baltimore, MD USA; 2 Department of Biomedical Engineering, Johns Hopkins School of Medicine, Baltimore, MD USA; 3 Kennedy Krieger Institute, Baltimore, MD USA; 4 Department of Neurology, Johns Hopkins School of Medicine, Baltimore, MD USA; 5 Department of Physical Medicine and Rehabilitation, Johns Hopkins School of Medicine, Baltimore, MD USA; 6 Kavli Neuroscience Discovery Institute, Johns Hopkins University, Baltimore, MD USA

**Keywords:** Human behaviour, Basal ganglia

## Abstract

Our assessments of effort are critically shaped by experiences of exertion. However, it is unclear how the nervous system transforms physical exertion into assessments of effort. Availability of the neuromodulator dopamine influences features of motor performance and effort-based decision-making. To test dopamine’s role in the translation of effortful exertion into assessments of effort, we had participants with Parkinson’s disease, in dopamine depleted (OFF dopaminergic medication) and elevated (ON dopaminergic medication) states, exert levels of physical exertion and retrospectively assess how much effort they exerted. In a dopamine-depleted state, participants exhibited increased exertion variability and over-reported their levels of exertion, compared to the dopamine-supplemented state. Increased exertion variability was associated with less accurate effort assessment and dopamine had a protective influence on this effect, reducing the extent to which exertion variability corrupted assessments of effort. Our findings provide an account of dopamine’s role in the translation of features of motor performance into judgments of effort, and a potential therapeutic target for the increased sense of effort observed across a range of neurologic and psychiatric conditions.

## Introduction

After a bout of physical activity, we assess how much effort we exerted and use this judgment to guide our decisions about future exertions. Previous work has shown that dopamine influences decisions between effortful exertion and reward by modulating reward value, without contributing to the estimation of effort cost^1–3^. However, increased dopaminergic availability has also been associated with increased effortful exertion and low-level features of motor performance^4–6^. These seemingly incongruous findings in the domains of effort-based decision-making and motor performance raise the question of how dopaminergic signaling might influence the translation of physical exertion into judgments of effort^7^.

Studies of the influence of dopaminergic availability on effort-based decision-making have shown that increased dopamine makes individuals more willing to exert effort for reward^3,5,8^. It has been suggested that this dopaminergic facilitation of effort-based decision-making is the result of dopamine alleviating motivational deficits by increasing reward sensitivity, rather than decreasing effort cost sensitivity^5,7,9^. These results point to dopamine’s influence on reward signaling that is dissociable from effort signaling.

In the context of motor performance, increased dopaminergic availability has been associated with increases in physical effort exertion and the initial velocity of exertion^4–6,10,11^. Relatedly, individuals with Parkinson’s disease (PD) exhibit a generalized slowness of movement (i.e., bradykinesia) that is exacerbated when they are withdrawn from dopaminergic medication. It has been proposed that dopaminergic availability does not influence the organization of movement per se, but rather the underlying motivation to initiate movement^10^. Despite dopamine’s influence on motor performance, it is unclear how such dopamine-mediated effects might impact judgments of physical effort and decisions to exert.

Here we investigated the influence of dopamine on the processes responsible for assessing effort levels following physical exertion. We hypothesized that variability in participants’ exertion would disrupt their assessments of effort, and dopamine would decrease both exertion variability and the extent to which variability inflates effort assessments. This hypothesis is motivated by studies showing that increased variability in motor output is associated with increased effort costs^6,12,13^. During effort assessments, such exertion variability may increase uncertainty in performance outcomes, and disrupt accurate effort assessment. Our hypothesized role of dopamine is based on studies that have found relationships between low-level features of motor performance and dopaminergic availability^4–6,10,11^. It has been suggested that dopaminergic modulation influences the signal-to-noise ratio in the nervous system, contributing to enhanced motor performance and sensory acuity^6,12,14^. With this in mind, we reasoned that dopamine could modulate variability in effort exertion (i.e., variability in motor output), which may lead to individuals’ more accurate assessments of effort.

**Fig. 1:**
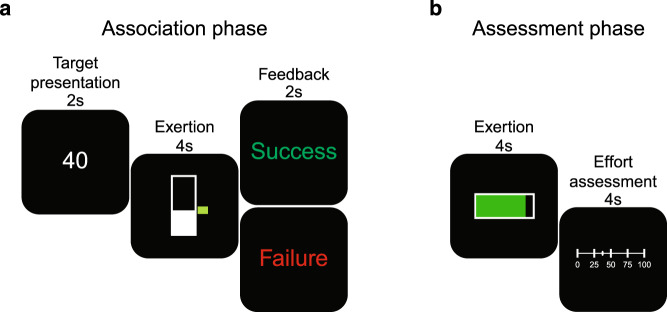
Experimental paradigm. **a** Association phase; Participants were trained to associate between grip force exerted on a handheld dynamometer and effort levels from 0 to 100 (80% of maximum voluntary contraction (MVC)). Each trial began with presentation of the target, followed by an effortful grip with real-time visual feedback of the exerted force represented as a bar that increased in height with increased exertion. A target zone was also presented, and participants were asked to maintain their exerted force within this target zone. The target zone turned green when exerted force was within the target and stayed red otherwise. Feedback of success or failure was provided at the end of each trial. **b** Assessment phase; Participants were instructed to exert an unknown amount of force and assess how much they exerted. On each trial, the full bar corresponded to a target effort level that was unknown to participants. Successfully achieving the effort target resulted in the bar turning from red to green. Following exertion, participants selected the effort level they believed they had exerted. Participants were not given feedback about accuracy of their effort assessment.

To test these hypotheses, we evaluated the influence of dopamine availability on assessments of effort in persons with PD. Participants performed a series of assessments of their physical exertion under conditions of decreased (OFF condition; dopaminergic medication withdrawal for at least 12 h) and increased (ON condition;1 h after medication intake) dopamine availability. The OFF and ON conditions were assessed on separate days. In each condition, participants were first trained to associate the grip force exerted on a hand-clench dynamometer with numeric effort levels between 0 and 100 (Fig. 1A). These effort levels were defined relative to a participant’s measured maximum voluntary contraction (MVC), obtained at the beginning of the experiment. Visual feedback of exertion was displayed on a vertical gauge that rose and fell in proportion to the amount of grip exertion. After the association phase, participants performed an effort assessment phase in which they produced exertion amounts on the dynamometer, and were presented with a horizontal gauge to fill, however explicit information as to the required effort units were not presented. Following exertion, participants were then asked to assess how much effort they exerted on a continuous scale from 0–100 (Fig. 1B). There were 48 trials in this assessment phase. Data from the assessment phase in both the OFF and ON conditions were analyzed to determine how differing amounts of exertion and variability in exertion performance were related to assessments of effort, and how dopaminergic state influenced effort exertion and judgment.

## Results

A total of 19 participants with idiopathic PD, diagnosed according to 2015 MDS Criteria^15^, were included in the analysis for this study (Supplementary Table 1). The mean disease duration of participants was 5 years 3 months (ranging from 1 year, 2 months - 20 years, 2 months). PD participants were tested on two days: ‘OFF’ – withdrawn from dopaminergic medication for at least 12 h (mean 13.5 h, ranging from 11–17 h); and ‘ON’ – testing session began one hour after their last dosage. The testing sessions were counterbalanced to avoid an effect of ordering and were not separated by more than 4 weeks (4.52 days, ranging between 1 day and 22 days). Participants were tested at the same time of day in both the ON and OFF conditions. To provide a reference for PD participants’ behavior we tested an additional 17 age-matched control participants.

**Fig. 2:**
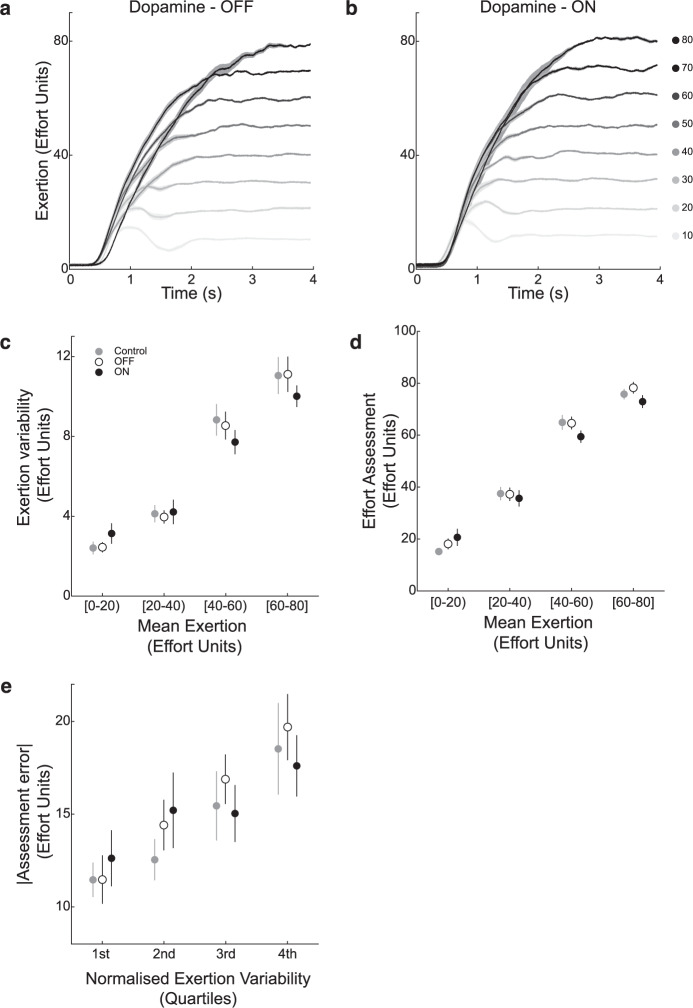
Behavioral data. Mean exertion profiles, during the assessment phase, for a representative participant in both the **a** Dopamine OFF and **b** ON conditions. All effort levels are presented in effort units, which were relative to participants’ maximum exertion. In both conditions, participants were able to exert to the target level and hold. The plots in Panels **c**–**e** were used for illustration purposes and not statistical inference, which was performed using mixed-effect linear models. (Control group – gray circles; OFF dopamine condition – open circles; ON condition – black circles). **c** Exertion variability as a function of mean exertion during the assessment phase. Exertion variability was calculated as the standard deviation of the last 3 s of exertion output. For illustration purposes, exertion variability was pooled in mean exertion bins of 20 effort units. There Error bars represent the standard error of the mean. When participants were in the Dopamine ON condition, they exhibited less of an increase in exertion variability as exertion increased, compared to OFF. Behavior for the control group matched participants in the OFF condition. **d** Effort assessment as a function of mean exertion during the assessment phase. Mean exertion was calculated as the average of the last 3 s of exertion output. For illustration purposes, effort assessments were pooled in mean exertion bins of 20 effort units. Error bars represent the standard error of the mean. Increased dopamine availability had a dampening effect on increases in effort assessment with exertion. Behavior for the control group matched participants in the OFF condition, and assessments were lower in the dopamine ON condition compared to controls. **e** Assessment errors increase with normalized exertion variability and this increase is more pronounced in the OFF-dopamine condition, compared to ON dopamine. For each trial, an assessment error metric was calculated by taking the difference between the assessed effort the mean exertion. A normalized exertion variability value was calculated by dividing exertion variability by mean exertion, which allowed performance during different levels of exertion to be evaluated in a unified model. For illustration, assessment errors and normalized exertion variability were pooled in quartiles of normalized exertion variability. Error bars represent the standard error of the mean. Increased exertion variability disrupted participants’ assessments of effort, and increased dopamine availability had a protective effect on the propensity for exertion variability to disrupt effort assessment. Behavior for the control group was not significantly different than behavior in the ON and OFF conditions.

During the effort assessment phase, in both the OFF and ON dopamine conditions, participants were able to exert effort at the target levels (Fig. 2A, B). A trial-by-trial analysis of the relationship between participants’ exertion variability (i.e., standard deviation of exertion following exertion ramp-up) and exertion showed that variability increased with increasing exertion for both control participants (Fig. 2C; Linear mixed model, β = 0.18311, *t* = 9.3352, df = 2634, *p* = 2.0745e−20) and those in the OFF and ON conditions (Fig. 2C; Supplementary Table 2; Linear mixed model, β = 0.17, *t* = 9.385, df = 1820, *p* = 1.8061e−20). This finding is consistent with previous studies of isometric force production which have shown that variability in motor output increases proportionally with the amount of exertion (i.e., signal-dependent noise)^16–18^. In the PD group, we found that dopamine availability interacted with the effect of mean exertion on exertion variability (Fig. 2C; Linear mixed model, β = −0.04, *t* = −2.25, df = 1820, *p* = 0.025). When participants were in the ON dopamine condition, they exhibited less of an increase in exertion variability as exertion increased, compared to the OFF condition. We found that control participants’ exertion variability matched participants in the OFF condition (Linear Mixed Model; β = −0.0030912, *t* = −0.11424, df = 2634, *p* = 0.90906, and the ON condition (Linear Mixed Model; β = −0.042854, *t* = −1.6234, df = 2634, *p* = 0.10462). These results align with our hypothesis of increased dopamine availability facilitating a reduction in exertion variability during effortful motor output.

During the assessment phase, we found that Control and (Fig. 2D; Linear mixed model, β = 1.226, *t* = 21.452, df = 2634, *p* = 3.1948e−94) PD participants in the ON and OFF conditions (Fig. 2D; Supplementary Table 3; Linear mixed model, β = 1.24, *t* = 17.49, df = 1820, *p* = 1.91e−63), recalled assessments of effort increased with their mean exertion. In the PD group, there was a significant interaction between dopamine treatment condition and mean exertion on assessments of effort (Linear mixed model; Fig. 2D; β = −0.23, *t* = −2.79, df = 1820, *p* = 0.005), indicative of increased dopamine availability having a dampening effect on increases in effort assessment with exertion. To further assess how dopaminergic availability was related to participants’ accuracy of effort assessment we examined the slope of the relationship between participants’ mean exertion and levels of assessment - perfect effort assessment would result in a slope of unity. In the dopamine OFF condition, the average slope was significantly greater than unity (β = 1.25 ± 0.07, t-stat = 3.36, df = 18, *p*-value = 0.0035), corresponding to overassessment of effort in a dopamine depleted state. In the dopamine ON condition, we failed to find a significant difference in slope from unity (β = 1.01 ± 0.09, t-stat = 0.11, df = 18, *p*-value = 0.91), suggesting that dopamine had a protective effect on effort assessment accuracy. Control participants’ effort assessments matched participants in the dopamine OFF condition (Linear Mixed Model; β = 0.0126, *t* = −0.139, df = 2634, *p* = 0.89), and assessments were lower in the dopamine ON condition compared to controls (Linear Mixed Model; β = −0.216, *t* = −2.083, df = 2634, *p* = 0.037). This comparison of the Control and PD groups show that these behavioral effects are generally related to dopaminergic availability/depletion, and not specifically related to dopamine interacting with PD disease symptomatology. Overall, these results suggest with participants translate their motor output into assessments of effort more accurately when in a state of increased dopamine availability.

To test our hypothesis regarding the influence of dopamine on the relationship between exertion variability and assessments of effort, we compared metrics related to exertion performance and participants’ ratings of their exertion. For each recall trial, we calculated a measure of normalized exertion variability as the standard deviation of exertion divided by the mean exertion on that trial (i.e., the coefficient of variation). This normalized metric allows us to account for the expected increase in variability with exerted effort (Fig. 2D) and evaluate how trial-to-trial variations in exertion variability are related to effort assessment errors (defined as the absolute difference between measured mean exertion and reported effort).

Overall, participants’ normalized exertion variability was related to the magnitude of their errors in effort assessment in both the Control (Fig. 2E; Linear mixed model; β = 0.25428, *t* = 3.804, df = 2634, *p* = 1.456e−04) and PD groups (Fig. 2E; Supplementary Table 4; Linear mixed model; β = 0.28, *t* = 4.9, df = 1820, *p* = 1.048e−06). During trials in which participants exhibited increased exertion variability, they had a more pronounced disagreement between their actual exerted effort and their assessed effort. This is consistent with the idea that increased exertion variability disrupted participants’ assessments of effort. Additionally, dopamine had a protective effect on the propensity for exertion variability to disrupt assessment errors (Fig. 2E; Linear mixed model; β = −0.14, *t* = −2.425, df = 1820, *p* = 0.015) – in the dopamine ON condition, increases in exertion variability had a less pronounced effect on discrepancies between actual exertions and assessments. We found that healthy-control participants’ assessment error matched participants in the dopamine OFF (Linear Mixed Model; β = 0.021935, *t* = 0.2513, df = 2634, *p* = 0.8016), and the dopamine ON conditions (Linear Mixed Model; β = −0.10564, *t* = −1.2864, df = 2634, *p* = 0.19843). These results show that dopamine influences the degree to which exertion variability impacts the transformation of exertion performance into assessments of effort.

**Fig. 3:**
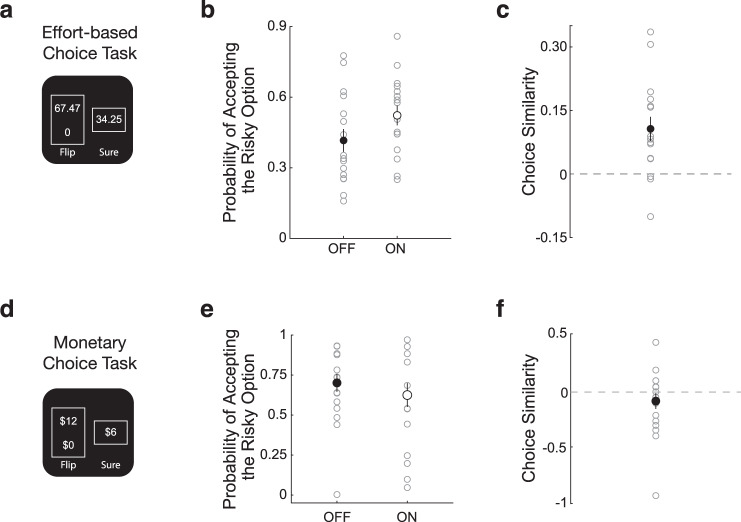
Effort-based and Monetary choice results. **a** During effort-based choice trials participants were presented a series of risky decisions that involved choosing between 2 options: exerting a low amount of effort with certainty (“sure”) or taking a gamble that could result in either a higher level of exertion or no exertion, with equal probability (“flip”). The effort amounts were presented on a 0–100 scale, on which participants were trained during an association phase before choice. An effort level of 0 corresponded to no exertion and 100 to 80% of a participant’s maximum exertion. Gambles were not realized after a choice. At the end of the Choice phase, to ensure that participants revealed their true preferences for effort, 10 choices were randomly selected and played out such that any effort required would need to be exerted before they completed the experiment. **b** Participants’ probability of accepting the risky effort option was significantly increased in the ON-dopamine condition compared to OFF-dopamine. Open circles show individual participant data, and the solid circle indicates the average probability of acceptance. Error bars indicate SEM. **c** Choice similarity metric comparing effort choices in the ON and OFF-dopamine conditions. Positive values indicate more risk-seeking behavior in the ON compared to OFF-dopamine condition. Open circles show individual participant data, and the solid circle indicates the average probability of acceptance. Error bar indicates SEM. **d** During monetary choice trials participants were presented with a series of risky decisions that involved choosing between 2 options: a small amount of monetary reward with certainty (“sure”) or taking a gamble that could result in either a larger amount of reward or no reward, with equal probability (“flip”). Gambles were not realized after a choice. At the end of the Choice phase, to ensure that participants revealed their true preferences for monetary reward, one choice was randomly selected and played out at the end of the experiment. **e** Participants’ probability of accepting the monetary risky effort option was not significantly different between the ON and OFF-dopamine conditions. Open circles show individual participant data, and the solid circle indicates the average probability of acceptance. Error bars indicate SEM. **f** Choice similarity metric comparing monetary choices in the ON and OFF-dopamine conditions. Positive values indicate more risk-seeking behavior in the ON compared to OFF-dopamine condition. Open circles show individual participant data, and the solid circle indicates the average probability of acceptance. Error bar indicates SEM.

Finally, if increased dopamine availability is related to judgments of effort, we would expect that dopamine may also have an influence on participants’ prospective decisions about effort. To test this hypothesis, we had participants perform an effort-based decision-making task, in isolation from reward, in both the ON and OFF dopamine conditions. During this task, participants made choices between a low amount of prospective effort with certainty; or an option involving either a prospective high-effort exertion or no exertion, with equal probability (Fig. 3A)^19–21^. This choice task exploited the theoretical equivalence between risk preferences and subjective valuation of effort in such a way that we could measure subjective valuation via the presentation of risky choices involving effort. Since participants performed the same set of choices in each condition, we were able to compute a choice similarity metric to directly compare how each participant’s decisions (and associated risk preferences) shifted in the ON compared to the OFF dopamine conditions. We have previously shown that inflated subjective effort cost valuation was accompanied by increased risk aversion for prospective effort^19,20^. Consistent with both effort assessments and prospective valuations of effort having lower aversive cost in conditions of higher dopamine availability, we found that participants were less risk averse for effort in the dopamine ON condition compared to OFF (Fig. 3B; single sample t-test on the difference between the log-odds, difference in log-odds = 0.49 ± 0.12, *t* = 4.02, df = 15, *p* = 0.0011). Since each effort choice was presented twice (ON condition and OFF condition), we could also examine the relationship between risk preference for identical choices. This analysis further confirmed that participants were less risk averse for effort in the dopamine ON condition compared to OFF (Fig. 3C; single sample t-test on the similarity metric; choice similarity metric = 0.11 ± 0.03, *t* = 3.76; df = 15; *p* = 0.0019). We also had participants perform a risky monetary decision-making task (Fig. 3D), separate from the effort-based decision-making task, and found no difference in their risk preferences between the ON and OFF conditions (Fig. 3E; single sample t-test on the difference between the log-odds, difference in log-odds = −0.078 ± 0.08, *t* = 1.10, df = 17, *p* = 0.1435 and choice similarity between conditions (Fig. 3F; single sample t-test on the similarity metric; choice similarity metric = −0.082 ± 0.31, *t* = = −1.1366; df = 17; *p* = 0. 0.2715). These results suggest that dopamine’s influence on risk preferences was specific to effort-based decision-making.

### Control analyses

It is important to note that tremor (involuntary oscillation of a limb about a joint) is one of the most common symptoms of PD^22^ and could be related to increased exertion variability. 12 of 19 participants in our study were classified as tremor dominant using their UPDRS scores. To test if increased exertion variability was generally related to dopaminergic modulation of Parkinsonian tremor, we evaluated participants’ exertion variability during the association phase of the experiment. We found that while exertion variability increased in proportion to mean exertion in the association phase (Linear mixed model, β = 0.034, *t* = 5.02, df = 1516, *p* = 5.73e−07), this variability was far lower than in the assessment phase, and the relationship between mean exertion and variability was not influenced by dopamine supplements in the association phase (Linear mixed model, β = −0.001, *t* = −0.23, df = 1516, *p* = 0.8). This illustrates that the dopaminergic modulation of exertion variability observed in the assessment phase was specific to the context of effort assessment and not generally related to the effect of Parkinsonian symptoms on motor performance. We also tested if dopamine had an influence on overall motor capacity by testing if there was a difference between participants’ MVC in the ON and OFF conditions and found no differences (paired t-test; *t* = 0.5; df = 1516; *p* = 0.32). These control analyses suggest that dopaminergic differences in effort assessment were not simply the result of the influences of dopamine on effort association or overall motor capacity, but rather on the performance of effort assessments specifically.

We also tested if there were relationships between UPDRS scores and changes in the relationships between parameter estimates with effort assessment and mean exertion (*n* = 19, *r* = 0.16, *p* = 0.51) and exertion variability (*n* = 19, *r* = −0.03, *p* = 0.91). We did not find significant relationships between dopamine-induced changes in behavior and clinical measures of Parkinson’s disease state.

To confirm that the treatment effects observed in the assessment phase were not solely a byproduct of the influence of dopamine on the development of associations between levels of exertion and units of effort, we analyzed participants’ performance during the association phase. We did not find a significant influence of dopamine availability on success/failure during the association phase, either overall (paired t-test; *t* = 0.534, df = 18, *p* = 0.6) or within individual target effort levels (Logistic mixed model; β = −0.0023, *t* = −0.25, df = 1516, *p* = 0.8).

A previous study of motor and motivational contributions of dopamine to exertion and decision found that increased dopamine availability was associated with a decreased time to reach peak exertion when compared to a dopamine-depleted state^5^. However, these differences in exertion response time were not modulated by motivational state. We did not find a significant difference in exertion ramp times between the dopamine-depleted and elevated states (Linear mixed model; β = −0.1117, *t* = −1.41, df = 1822, *p* = 0.16). To confirm that the dopaminergic effects on effort assessments we observed were not simply the result of differences in exertion ramp time, we performed a regression between participants exertion ramp times and effort assessments. We did not find a significant interaction between ramp times in the ON and OFF dopamine conditions and effort assessments (Linear mixed model; β = 0.032, *t* = 1.46, df = 1820, *p* = 0.14). These findings suggest that exertion ramp time alone did not influence effort assessments.

## Discussion

Here we show that increased dopamine availability reduces variability in effortful exertion and that these decreases in variability are related to increased accuracy of effort assessment. These findings show that dopamine has a protective effect on assessments of effort by reducing variability in motor performance. Our results align with previous theoretical^23–25^ and experimental^6,12^ accounts that have suggested that dopamine availability influences features of motor performance and facilitates the more salient encoding of stimuli. Our finding of improved accuracy in effort assessment in conditions of increased dopamine availability aligns with previous studies of effort-based choice, which found that dopamine increased the likelihood of selecting high-effort options, consistent with decreased levels of effort aversion^3,5,8,26,27^. However, these previous studies focused on dopaminergic function during stimulus discrimination and effort/reward trade-offs and did not consider how dopamine impacted the more basic functions of subjective effort assessment. Our results go beyond these studies by providing a framework within which dopamine influences fundamental parameters of exertion (i.e., exertion variability), and higher-level cognitive assessments of effort. While previous work has shown that dopamine mitigates effort cost through incentivization of reward, here we demonstrate the role of dopamine in influencing assessments of effort and associated effort-based decision-making.

Motor variability can have positive effects on motor learning by facilitating exploration and improving learning^28,29^, and negative effects by reducing the accuracy of intended movements^30^. Movement while in a dopamine-depleted state is more variable, especially as the magnitude of the required movement increases^6,30–32^. Here we demonstrate that increased motor variability, within the framework of sustained force production, influences assessments of effort. We did not observe dopamine-modulated differences in exertion variability with effort when the target effort was explicitly presented to participants in the association phase. This suggests that the differences during the effort assessment phase may arise from dopamine’s influence on the transfer of movement parameters to assessments of effort. Uncertainty arising from this variability may affect the judgment of task performance, leading to inflations in assessment. We have recently showed, in healthy participants, that increased variability serves as an added cost that inflates assessments and valuation of effort^13^.

Increased feelings of effort are likely to influence the boundary between effort-reward trade-offs, such that the reduced willingness to exert for reward in a dopamine-depleted state may be driven by inflated estimates of effort valuation in addition to decreases in reward sensitivity. Our finding that dopamine depletion increases variability in motor performance and risk aversion for effort, isolated from reward, aligns with previous findings of an increased reluctance to allocate physical effort in individuals with PD^6^. In this framework, it is possible that dopamine directly effects subjective assessments of exerted physical effort, which influences risk preferences for prospective decisions about effort exertion.

Another potential interpretation of our data is that dopamine availability could influence the accuracy with which an individual exerts effort, making exertions feel more difficult, and in turn, effort more costly. If this were the case dopamine availably would directly impact the efficiency of the motor system, to eventually influence effort assessments. Our experimental paradigm was not designed to differentiate between dopamine’s influence on effort exertion, assessment, or an interaction between the two. In the future it will be important to design studies of effort assessment that are able to dissociate the influence of dopamine on these intertwined variables.

Errors in effort assessment could arise from inaccurate estimates of exerted effort, or from an incorrect mapping between force production and effort levels during association. It has been shown that when learning about effort/reward trade-offs, reward and effort prediction errors are commonly encoded in the dopaminergic midbrain, suggesting a common neural substrate that underlies the generation of effort and reward associations^33^. We did not find a significant relationship between dopamine and performance during the association phase, and the behavioral effects of dopamine availability were best described by effort assessment performance. However, our experiment was not specifically designed to dissociate effort-based learning and valuations of effort. Experiments that explore individuals’ assessments of effort throughout the course of effort-based learning could elucidate the interactions between the generation of effort associations and valuation.

It is also important to note that when comparing data from the control and PD groups, we only found a significant difference between the control group and participants in the ON condition, in the effort assessment analysis. The lack of significance in the other analyses was likely due to our experiment being underpowered to detect significant differences between the control and patient groups, while comparisons between the ON and OFF dopamine conditions relied on more statistically powerful pairwise analyses. Essentially, it was difficult to detect significant differences between the control and PD groups because we were making population level comparisons between groups, while between the PD ON and OFF conditions we were able to use within participant comparisons.

In summary, our study shows that dopamine availability influences the transformation of physical exertion into assessments of effort by modulating the extent to which exertion variability impacts judgments of effort. This work begins to bridge the gap in understanding how dopamine influences the translation of motor performance into assessments of physical effort.

## Methods

### Participants

The protocol was approved by the Johns Hopkins University Institutional Review Board, and all participants provided written informed consent. A total of 24 persons with Parkinson’s disease participated in this study and were pre-screened to exclude those with any other neurological disorders. Five participants were excluded from the analysis for a variety of reasons. One participant did not express willingness to return to complete the study, one participant reported having an implanted deep brain stimulator (only after the first session was completed), and one participant had pronounced tremor that interfered with the ability to control a mouse to report exerted effort, and two participants had severe cognitive difficulties and were unable to fully follow the experiment instructions. The final cohort was comprised of a total of 19 participants (Supplementary Table 1). PD participants were tested on two days: ‘OFF’ –withdrawn from dopaminergic medication for at least 12 h; and ‘ON’ – testing session began one hour after their last dosage. The testing sessions were counterbalanced to avoid the effect of ordering and were not separated by more than 4 weeks.

To provide a reference for PD participants’ behavior we tested an additional 17 age-matched control participants (Supplementary Table 1). Control participants were recruited from the local Baltimore community, screened for the absence of acute depression and dementia (Hamilton Depression Rating scale and Mini Mental State Examination), and were not taking any dopaminergic medication at the time of the study. Control participants were tested on the behavioral paradigm once.

### Experiment setup

Participants were presented with visual cues using custom MATLAB (Mathworks 2018a) code utilizing PsychToolBox libraries^34^. Participants expended grip force effort by squeezing a hand clench dynamometer (HD-BTA, Vernier Inc., Beaverton, OR, Acquisition frequency – 2000 Hz) and were asked to assess the level of exerted effort using a sliding scale presented on a computer screen in front of them. During experiments, signals from this dynamometer were sent to our software for real-time visual feedback of participants’ exertion. To record participants’ assessments of effort and choices, we collected keystrokes from a standard computer keyboard. Exertions with the dynamometer were performed with participants’ dominant hand, and keyboard selections were made with the nondominant hand.

### Effort assessment paradigm

Before beginning the experiment, participants were told that they would receive a show-up fee of $15/h, and that this fee was not dependent on their performance over the course of the experiment. The effort assessment paradigm was identical to those we have previously used^19,20^.

On each day of the experiment participants began by exerting their maximum voluntary contraction (MVC). Participants were asked to squeeze the dynamometer as hard as they could for four seconds, on three consecutive trials, and each trial was followed by a period of rest. A participant’s MVC was defined as the maximum force exerted over all trials.

Participants were next evaluated in an association phase where they were trained to associate the force they exerted against the dynamometer with different levels of effort (Fig. 1A). Effort levels were presented on a scale that ranged from 0 (no exertion) to 100 (80% of a participant’s MVC). Participants performed association trials that ranged from 10–80 effort units, in increments of 10 effort units. Each association trial began with the presentation of the numeric target level for 1.5 s. This was followed by an exertion task in which visual feedback was provided in the form of a vertical gauge whose level rose and fell in proportion to grip effort. The gauge had a range from 0–100 effort units. The bar was empty to begin with and filled up as participants exerted grip effort. For each trial, a target zone (+/−5 effort units) was also provided beside this bar. This target zone was red to begin with and turned green when participants exerted effort levels within this range. Participants were instructed to squeeze and reach the target zone as quickly as possible and sustain the effort level for the duration of the exertion task (4 s). At the end of the exertion task participants were informed whether they were successful or failed at exerting the required effort. A trial was considered successful if participants remained in the target zone for at least 2.67 s of the exertion phase. During the association phase participants were presented with effort levels in blocks of 5 trials, and each effort level block was presented in a randomized order to prevent any ordered effect of muscle fatigue with time. To minimize fatigue, 1–4 s rest was provided between trials in a training block, and 20 s rest was provided between training blocks.

Participants next performed an effort assessment phase to determine how they generated assessments of their levels of exertion, and if dopamine influenced these assessments. Participants were tested on each of the previously trained effort levels (10–80 effort units, in increments of 10 effort units). Each assessment trial began with a black horizontal effort gauge that participants were instructed to fill completely, and hold, by exerting on the force transducer. Participants were given 4 s to exert, and as for the association phase, instructed to squeeze to reach the end of the gauge as quickly as possible and sustain the effort level for the duration of the task (4 s). Unlike the association phase, the end of the effort gauge did not represent effort level 100. Instead, it represented the target effort level selected from the range 10–80 effort units, incremented by 10 effort units. In this way participants were not given explicit feedback of the units of effort associated with their exertion. Following exertion, participants were presented with a number line that ranged from 0 to 100 and instructed to select the effort level that they believed that they had just exerted. The cursor was moved along a number line using a mouse to change the value and clicking the left mouse button to select an effort level. Participants were tested on 48 trials, with 6 trials for each effort level from 10 to 80. These effort levels were presented in a random order, and no feedback was provided to the participants about the accuracy of their effort assessments. Upon selection, a fixation cross appeared on the screen for 1–4 s to provide rest between trials. A longer rest of 20 s was provided midway through the phase.

#### Effort choice task

After the assessment phase, participants performed a series of effort gambles and the choices from these gambles were used to characterize participants’ risk preferences and associated subjective preferences for effort. We have used this paradigm in a series of previous studies ^19–21^ Before being presented with the effort gambles, participants were told that 5 of their decisions would be selected at random at the end of the experiment and that they would have to remain in the testing area until they achieved the exertions required. This was done to ensure that participants were properly incentivized on each trial.

A single effort gamble involved of choosing between two options shown on the screen under a time constraint (4 s): exerting a low amount of force (*f*_s_) with certainty; and a risky option that could result in either high exertion (*f*_g_) or no exertion, with equal probability (Fig. 3A). The effort levels were presented on the 0–100 scale that participants trained on during the association phase. Participants made their choices by pressing one of two buttons on a standard keyboard. Gambles were not resolved after choice, and participants did not perform the exertion task during this phase of the experiment. Effort gambles (170 in total) were presented consecutively in a randomized order. The effort amounts for the choice set were designed to accommodate a range of effort preferences and we have used this choice set to examine effort valuation in several previous studies^19–21^. Participants were encouraged to make a choice on every trial; however, there was no penalty for failing to decide within the 4-second time window.

After the choices had been completed, the computer selected 5 of the trials at random to be implemented. The outcomes of the selected trials, and only those trials, were implemented. In this way, participants did not have to worry about spreading their effort exertion over all of their trials. Participants were instructed that the experiment would not be completed, and they were to remain in the testing area, until they achieved the chosen effort levels.

This effort choice paradigm exploits the theoretical equivalence between risk preferences and subjective valuation to measure subjective valuation via the presentation of risky choices, a widely accepted practice in economics and decision neuroscience^35,36^. Moreover, choice prospects only involved varying amounts of physical effort (i.e., no prospective rewards were involved), which allowed us to isolate processes related to effort valuation, separate from the effects of reward valuation or effort/reward trade-offs.

Participants with PD performed the effort choice task on both days of the experiment, and those in the control group performed the effort choice task once.

#### Monetary choice task

At the end of the experiment participants were also tested on their monetary risk attitude using a series of gambles that included only financial gains. In each trial, each participant was presented with the choice either to accept a safe option (i.e., a variable sure monetary amount) or to play a risky gamble (i.e., flip a coin to receive a larger amount of money or get nothing). The sure amount ranged from $1 to $12. Corresponding gambles ranged from $2 to $30. A total of 20 trials were presented. At the end of the experiment a trial was randomly selected, and a payment was made according to the participants’ decision and a random outcome. These exact choices have been used to elicit risk aversion in several previous studies^37–39^.

### Exertion metrics

To analyze participants’ exertion performance during the assessment and association phases we calculated metrics of performance for the final 3 s of the 4 s exertion segment (henceforth referred to as the target period). We excluded data from the first second of trials to remove variability in performance arising from different response times. Exertion performance metrics were expressed in effort units.

We calculated participants’ mean exertion (*ME*) during the target period. To evaluate exertion variability (*EV*) we calculated the standard deviation of participants’ exertion during the target period. We also calculated a normalized exertion variability (*EVN*) metric in which we divided the standard deviation of participants’ exertion during the target period by their mean exertion (i.e., the coefficient of variation of the exertion variability). The normalized variability controlled for the relationship between increasing levels of exertion and exertion variability so that we could evaluate how trial-to-trial variations in exertion variability were related to assessments of effort.

### Models

We used mixed-effect linear models to assess the relationship between participants’ trail-to-trial variance in exertion performance was related to trial-to-trial variance in their ratings of exertion, and how dopamine influenced these relationships. These analyses were performed using MatabR2022a, using the *fitglme* function in the Statistics and Machine Learning Toolbox. We tested relationships between *ME*, *EV*, and effort assessments (*EA*). When comparing treatment conditions (Dopamine ON vs OFF), we used a mixed effects model with participant as a random effect, and the treatment condition, appropriate covariate (mean exertion, exertion variability, and normalized exertion variability), and the interaction between these factors as fixed effects. When comparing two populations (Healthy controls vs Persons with PD), we used a mixed effects model with participant as a random effect nested under population, and fixed factors as described above.

1 $$\begin{array}{l}EA_i = \beta _1 \ast ME_i + \beta _2 \ast Treatment_i + \beta _3 \ast \left( {ME \ast Treatment} \right)_i\\ + \left( {ME_i + Treatment_i + \left( {ME \ast Treatment} \right)_i} \right)\left| {Subject_j} \right.\end{array}$$

To evaluate the accuracy of participants assessment of effort we computed the above model individually for each subject, in each treatment condition, and compared the population slope to using a single sample t-test unity (perfect effort assessment would correspond to a slope of 1).

2 $$\begin{array}{l}EV_i = \beta _1 \ast ME_i + \beta _2 \ast Treatment_i + \beta _3 \ast \left( {ME \ast Treatment} \right)_i\\ + \left( {ME_i + Treatment_i + \left( {ME_i \ast Treatment} \right)_i} \right)|Subject_j\end{array}$$

Where *i* denoted trial number, *j* denoted subject number (1–19), and *Treatment* was a factorial variable (0 – Control, 1 – Dopamine-OFF, 2 – Dopamine-ON). This analysis was performed for both the Association and Assessment phases.

We also computed a model to test how trial-to-trial discrepancies between participants’ effort assessments and their mean exertion (assessment error; AE: the absolute difference between mean exertion and effort assessments) were related to their normalized exertion variability (*EVN*). This model allowed us to test if variability in exertion contributed to participants errors in effort assessment.

3 $$\begin{array}{l}AE_i = \beta _1 \ast EVN_i + \beta _2 \ast Treatment_i + \beta _3 \ast \left( {EVN \ast Treatment} \right)_i\\ + \left( {EVN_i + Treatment_i + \left( {EVN \ast Treatment} \right)_i} \right)|Subject_j\end{array}$$

#### Control analyses

To confirm that the treatment effects observed in the assessment phase were not a byproduct of the influence of dopamine on the development of associations between levels of exertion and units of effort, we analyzed participants’ performance during the association phase. We determined the effect of dopamine availability on the relationship between the presented effort level, and being successful in performing the required exertion at that level, in the association phase:

4 $$\begin{array}{l}\log \left( {\frac{{{\it{Probability}}\,{\it{of}}\,{\it{success}}\,{\it{at}}\,{\it{target}}\,{\it{level}}}}{{{\it{Probability}}\,{\it{of}}\,{\it{failure}}\,{\it{at}}\,{\it{target}}\,{\it{level}}}}} \right)_i = \beta _1 \ast Target_i + \beta _2 \ast Treatment_i\\ \, + \beta _3 \ast \left( {Target \ast Treatment} \right)_i + \left( {Target_i + Treatment_i + \left( {Target \ast Treatment} \right)_i} \right)\left| {Subject_j} \right.\end{array}$$

To test how initial exertion performance was related to dopamine availability, and participants’ subsequent assessments of effort, we performed a regression between participants’ trial-to-trial exertion ramp durations and effort assessments.

5 $$\begin{array}{l}EA_i = \beta _1 \ast Ramp\,Time_i + \beta _2 \ast Treatment_i + \beta _3 \ast \left( {Ramp\,Time \ast Treatment} \right)_i\\ \, + \left( {Ramp\,Time_i + Treatment_i + \left( {Ramp\,Time \ast Treatment} \right)_i} \right)\left| {Subject_j} \right.\end{array}$$

Participants’ exertion ramp time $RampTime_i$, on each trial, was defined as the duration between their reaction time and the time at which they reached the exertion target window for the first time.

### Choice similarity metric

To assess how risk attitudes (and associated subjective valuation) for effort and money change between treatment conditions, we compared choices between conditions by computing a choice similarity metric. This metric is model-free and does not assume an effort utility function or require the fitting of a model to the behavioral data. Since each effort/monetary gamble was presented twice (once per treatment condition), it is possible to examine if choice behavior for identical effort options changed between treatment conditions. To generate this metric, a value of 0 was assigned to a choice trial in the ON condition if the participant made the same choice as in the OFF condition; +1 was assigned to a choice if the participant accepted a gamble in the ON condition that they rejected in the OFF condition (i.e., more risk seeking behavior); and −1 was assigned if the participant rejected a gamble in the ON condition that they accepted in the OFF condition (i.e., more risk averse behavior). In the analysis of effort choice, we excluded participants (*n* = 3) who had an acceptance rate below 5% or greater than 95% as we took this to indicate a lack of evaluating effort options, and merely choosing the same option on every trial. We calculated the monetary choice similarity metric for the same group of participants as for the effort similarity metric.

### Reporting summary

Further information on research design is available in the Nature Research Reporting Summary linked to this article.

## Supplementary information

Supplemental Material

Reporting Summary

## Data Availability

The source data underlying are available for download at https://osf.io/d9kuh/.

## Code availability

All analysis code will be available upon request.
