# Supplementary material for: Dopamine facilitates the translation of physical exertion into assessments of effort
Source: NPJ Parkinsons Dis. 2023 Apr 1;9:51. doi: 10.1038/s41531-023-00490-4 (PMC10067851; doi:10.1038/s41531-023-00490-4)
Supplement: Supplementary file 1 — Supplemental Material [file 41531_2023_490_MOESM1_ESM.pdf]

Supplementary Information for:

Dopamine Facilitates the Translation of Physical Exertion into Assessments of Effort

Purnima Padmanabhan[1], Agostina Casamento-Moran[2], Aram Kim[2], Anthony J. Gonzalez[3], Alexander Pantelyat[4], Ryan T. Roemmich[3,5], and Vikram S. Chib[2,3,6*]

[1]Department of Neuroscience, Johns Hopkins School of Medicine

[2]Department of Biomedical Engineering, Johns Hopkins School of Medicine

[3]Kennedy Krieger Institute

[4]Department of Neurology, Johns Hopkins School of Medicine

[5]Department of Physical Medicine and Rehabilitation, Johns Hopkins School of Medicine

[6]Kavli Neuroscience Discovery Institute, Johns Hopkins University

*Correspondence and requests for materials should be addressed to:

Vikram S. Chib
707 North Broadway
Baltimore, MD 21205, USA
443-923-2716
vchib@jhu.edu

Abbreviated Title: Dopamine Influences Assessments of Effort

	PD-OFF	PD-ON	Control
Age	66.94(1.7)	-	64.94(1.83)
Sex	10M/9F	-	9M/8F
Handedness	18R/1L	-	13R/4L
UPDRS (Section-III)	34.94(2.82)	30(2.47)*	N/A
Hoehn & Yahr	2.11(0.07)	2.06(0.04)	N/A
Hamilton Depression Rating	8.94(1.05)	7.94(1.23)#	3.93(0.88)
Mini Mental State Exam	28.17(0.45)	28.28(0.35)#	29.44(0.16)

Supplementary Table 1: Participant Demographics. Numbers in parentheses are standard deviations. *p<0.05 between PD-ON and PD-OFF. #p<0.05 (corrected for multiple comparisons) between healthy controls and PD.

Parameter Predicting Exertion Variability	Estimate	Significance
Mean Exertion	1.24 ± 0.07	0.04
Dopamine Availability (ON/OFF)	0.06 ± 0.03	1.91 E-63
Mean Exertion × Dopamine Availability	-0.23 ± 0.08	0.005

Supplementary Table 2: Parameter estimates and significance for the model illustrated in Figure 2C.

Parameter Predicting Effort Assessment	Estimate	Significance
Mean Exertion	0.18 ± 0.02	0.08
Dopamine Availability (ON/OFF)	0.01 ± 0.008	1.81 E-20
Mean Exertion × Dopamine Availability	-0.04 ± 0.02	0.02

Supplementary Table 3: Parameter estimates and significance for the model illustrated in Figure 2D.

Parameter Predicting |Assessment Error|	Estimate	Significance
Normalized Exertion Variability	0.24 ± 0.03	3.90E-14
Dopamine Availability (ON/OFF)	0.04 ± 0.01	0.001
Exertion Variability × Dopamine Availability	-0.15 ± 0.04	0.0001

Supplementary Table 4: Parameter estimates and significance for the model illustrated in Figure 2E.
