# Supplementary material for: Dopamine facilitates the translation of physical exertion into assessments of effort
Source: NPJ Parkinsons Dis. 2023 Apr 1;9:51. doi: 10.1038/s41531-023-00490-4 (PMC10067851; doi:10.1038/s41531-023-00490-4)
Supplement: Supplementary file 2 — Reporting Summary [file 41531_2023_490_MOESM2_ESM.pdf]

nature portfolio

Corresponding author(s): Chib, Vikram

Last updated by author(s): Nov 15, 2022

Reporting Summary

Nature Portfolio wishes to improve the reproducibility of the work that we publish. This form provides structure for consistency and transparency in reporting. For further information on Nature Portfolio policies, see our Editorial Policies and the Editorial Policy Checklist.

Statistics

For all statistical analyses, confirm that the following items are present in the figure legend, table legend, main text, or Methods section.

n/a	Confirmed	
☐	☒	The exact sample size (*n*) for each experimental group/condition, given as a discrete number and unit of measurement
☐	☒	A statement on whether measurements were taken from distinct samples or whether the same sample was measured repeatedly
☐	☒	The statistical test(s) used AND whether they are one- or two-sided<br>*Only common tests should be described solely by name; describe more complex techniques in the Methods section.*
☐	☒	A description of all covariates tested
☐	☒	A description of any assumptions or corrections, such as tests of normality and adjustment for multiple comparisons
☐	☒	A full description of the statistical parameters including central tendency (e.g. means) or other basic estimates (e.g. regression coefficient) AND variation (e.g. standard deviation) or associated estimates of uncertainty (e.g. confidence intervals)
☐	☒	For null hypothesis testing, the test statistic (e.g. *F*, *t*, *r*) with confidence intervals, effect sizes, degrees of freedom and *P* value noted<br>*Give P values as exact values whenever suitable.*
☒	☐	For Bayesian analysis, information on the choice of priors and Markov chain Monte Carlo settings
☐	☒	For hierarchical and complex designs, identification of the appropriate level for tests and full reporting of outcomes
☐	☒	Estimates of effect sizes (e.g. Cohen's *d*, Pearson's *r*), indicating how they were calculated

Our web collection on statistics for biologists contains articles on many of the points above.

Software and code

Policy information about availability of computer code

Data collection	The experimental task was programmed in Matlab 2018a using Psychtoolbox-3 (version 3.0.11).
Data analysis	Analyses were performed using MatabR2022a, using the fitglme function in the Statistics and Machine Learning Toolbox

For manuscripts utilizing custom algorithms or software that are central to the research but not yet described in published literature, software must be made available to editors and reviewers. We strongly encourage code deposition in a community repository (e.g. GitHub). See the Nature Portfolio guidelines for submitting code & software for further information.

Data

Policy information about availability of data

All manuscripts must include a data availability statement. This statement should provide the following information, where applicable:

- Accession codes, unique identifiers, or web links for publicly available datasets
- A description of any restrictions on data availability
- For clinical datasets or third party data, please ensure that the statement adheres to our policy

All unique materials and raw data will be readily available from the authors or freely available online.

Human research participants

Policy information about studies involving human research participants and Sex and Gender in Research.

Reporting on sex and gender	Participants self-reported their gender. 10 male and 9 female participants with Parkinson's Disease took part in the study. 9 male and 8 female participants with Parkinson's Disease took part in the study.
Population characteristics	See 'Behavioural & social sciences study design' section below.
Recruitment	Parkinson's participants were recruited from the Johns Hopkins Parkinson's Disease and Movement Disorders Center. Control participants were recruited from the local Baltimore community.
Ethics oversight	Johns Hopkins University Institutional Review Board

Note that full information on the approval of the study protocol must also be provided in the manuscript.

Field-specific reporting

Please select the one below that is the best fit for your research. If you are not sure, read the appropriate sections before making your selection.

☐ Life sciences ☒ Behavioural & social sciences ☐ Ecological, evolutionary & environmental sciences

For a reference copy of the document with all sections, see nature.com/documents/nr-reporting-summary-flat.pdf

Behavioural & social sciences study design

All studies must disclose on these points even when the disclosure is negative.

Study description	This study involved the collection of quantitative behavioral data.
Research sample	A total of 24 persons with Parkinson's disease participated in this study and were pre-screened to exclude those with any other neurological disorders. PD participants were tested on two days: 'OFF' –withdrawn from dopaminergic medication for at least 12 hours; and 'ON' – testing session began one hour after their last dosage. The testing sessions were counterbalanced to avoid an effect of ordering and were not separated by more than 4 weeks.<br><br>To provide a reference for PD participants' behavior we tested an additional 17 age- matched control participants. Control participants were screened for the absence of acute depression and dementia (Hamilton Depression Rating scale and Mini Mental State Examination), and were not taking any dopaminergic medication at the time of the study. Control participants were tested on the behavioral paradigm once.
Sampling strategy	Participants in the Parkinson's and Control groups took part in the experiment on a first-come, first-served basis with considerations for individual schedules and booking constraints. This sample size was motivated based on previous studies of effort-based choices by our group, and others.
Data collection	The experimenter was present throughout every step of data collection.<br><br>Presentation of visual stimuli and acquisition of behavioral data were achieved using custom MATLAB (http://www.mathworks.com) scripts implementing the PsychToolBox libraries. Participants' responses were recorded by keyboard button press.<br><br>An hand clench dynamometer (TSD121B-MRI, BIOPAC Systems, Inc., Goleta, CA) was used to record grip force effort exertion. During experiments, signals from this sensor were sent to our custom designed software for visual real-time feedback of participants' exertion. Effort exertion was performed while participants held the force transducer.
Timing	2019/01/15 - 2020/03/01
Data exclusions	Five participants were excluded from the analysis for a variety of reasons. One participant did not express willingness to return to complete the study, one participant reported having an implanted deep brain stimulator (only after the first session was completed), and one participant had pronounced tremor that interfered with the ability to control a mouse to report exerted effort, and two participants had severe cognitive difficulties and were unable to fully follow the experiment instructions. The final cohort was comprised of a total of 19 participants with Parkinson's Disease.<br><br>No control participants were excluded.
Non-participation	One participant in the Parkinson's Group did not express willingness to return to complete the study.
Randomization	Participants were either assigned to a Parkinson's or Control Group. Individuals in the Parkinson's Group were randomly assigned to

Randomization	the ON and OFF dopamine conditions first.

Reporting for specific materials, systems and methods

We require information from authors about some types of materials, experimental systems and methods used in many studies. Here, indicate whether each material, system or method listed is relevant to your study. If you are not sure if a list item applies to your research, read the appropriate section before selecting a response.

Materials & experimental systems

n/a	Involved in the study
☒	☐ Antibodies
☒	☐ Eukaryotic cell lines
☒	☐ Palaeontology and archaeology
☒	☐ Animals and other organisms
☒	☐ Clinical data
☒	☐ Dual use research of concern

Methods

n/a	Involved in the study
☒	☐ ChIP-seq
☒	☐ Flow cytometry
☒	☐ MRI-based neuroimaging
